# Unitization Based Memory Enhancement in Younger and Older Adults

**DOI:** 10.5334/joc.457

**Published:** 2025-07-31

**Authors:** Joshua Kah Meng Khoo, Roni Tibon

**Affiliations:** 1 School of Psychology, University Park, The University of Nottingham, Nottingham, NG7 2RD, UK

**Keywords:** Aging, Episodic Memory, Familiarity, Recollection, Unitization

## Abstract

Memory for episodic associations declines with ageing due to decreased recollection abilities. Unitization—the encoding of multiple items as one integrated entity—has been shown to support familiarity-based retrieval that is independent of recollection and is relatively preserved in healthy ageing. Accordingly, unitization has been proposed as a promising strategy to attenuate age-related associative deficits, but evidence regarding its utility was lacking. The current study aimed to establish unitization as a viable mnemonic strategy. First, to ensure that unitization can attenuate the age-related associative deficit for initially unrelated materials, top-down unitization was used. Namely, participants were given an initially unrelated word pair in the context of either a definition which allows the words to be encoded as a unitized compound or a sentence in which the words are encoded as separate entities. Second, to ensure that unitization can be used as a self-initiated strategy, participants also completed the task by generating their own binding information (definitions/sentences). As expected, a unitization effect had emerged, such that associative memory was enhanced following definition encoding. However, this effect only occurred when binding information was provided. Additionally, a general memory advantage for the self-generation condition had emerged, but this was (generally) similar across unitization conditions and age groups. Taken together, the results show that unitization can be used as a mnemonic strategy under certain conditions, and highlight additional steps that should be taken before it can be effectively used beyond lab settings.

## Introduction

With the increasing proportion of older adults in the worldwide population (Beard et al., 2016), there is a pressing need to understand the cognitive changes associated with ageing, and to develop mechanisms to attenuate age-related cognitive decline. In recent years, major research efforts have been dedicated to the alleviation of age-related decline in memory. Indeed, memory problems are among the most common complaints as people grow older and can have far-reaching effects on the individual’s functioning and well-being (e.g., Jetten et al., 2010; Verhaeghen et al., 2000).

Whilst some aspects of the human memory are severely affected by ageing, others show a more attenuated decline or might even be preserved, owing to the notion that memory consists of different systems which subserve different functionality and supported by distinct brain regions (e.g., Gabrieli, 1998; Squire et al., 1993). The episodic memory system is one system known to be severely affected by cognitive ageing, but even within this system, some aspects are more affected than others. One distinction within the episodic memory system is between item memory, which involves remembering individual items such as a word or an object, and associative memory which requires remembering the relationship between multiple items. The widely supported associative-deficit hypothesis (ADH) postulates that the age-related decline in episodic memory stems at least partially from deficits in the ability to encode and retrieve associations (Bender et al., 2010; Brubaker & Naveh-Benjamin, 2014; Naveh-Benjamin, 2000; Naveh-Benjamin et al., 2003, 2007; Old & Naveh-Benjamin, 2008). This deficit is further characterized by relatively preserved memory for single items alongside a sharp decline in associative memory in older compared to young adults. It is widely evident in associative recognition tests, in which participants are required to discriminate between intact (studied) and recombined (studied items in new combinations) stimulus pairs, and has been demonstrated across a wide range of materials (see Old & Naveh-Benjamin, 2008 for Meta-analysis).

This difficulty that older adults experience in recognizing episodic associations is often explained in the context of the commonly accepted dual-process theory of episodic recognition (e.g., Yonelinas, 2002; Yonelinas et al., 2010). This theory posits that recognition is supported by two separable processes: familiarity and recollection. Familiarity refers to the feeling of having previously encountered something or someone without retrieval of additional information, while recollection provides additional contextual details about that encounter. Because contextual details are inherently associative (i.e., depending on the links made between items and their surrounding spatiotemporal context), it was traditionally agreed that while recognition of single items can be supported by both recollection and familiarity, in associative recognition tasks recollection is required for the retrieval of novel episodic associations, and that such associative memory is not accessible via familiarity (e.g., Donaldson & Rugg, 1998; Hockley & Consoli, 1999; Yonelinas, 1997). As age-related deficits in episodic memory are mainly observed for associative (but not item) recognition, it was asserted that they reflect impaired recollection, contrasted with relatively preserved familiarity (e.g., Davidson & Glisky, 2002; Friedman, 2013; Koen & Yonelinas, 2016).

Despite the abovementioned traditional view that associative recognition relies solely on recollection, more recently it was suggested that in some situations it might be alternatively (or conjointly) supported by familiarity. In particular, it was noted that when the to-be-associated memoranda are perceived and encoded as a single unit entity—that is, bound together to form a unitized representation—familiarity can contribute to their associative retrieval (e.g., Graf & Schacter, 1989; Han et al., 2023; Jäger et al., 2006; Jäger & Mecklinger, 2009; Li et al., 2019; Parks & Yonelinas, 2015; Quamme et al., 2007; Rhodes & Donaldson, 2007, 2008; Tibon, Ben-Zvi, et al., 2014; Tibon et al., 2012; Tibon, Gronau, et al., 2014; Zheng et al., 2015). This notion is supported by a growing body of evidence (for review see Yonelinas et al., 2010), including intact memory for unitized associations in healthy older adults (Bastin et al., 2013; Delhaye et al., 2018; Delhaye & Bastin, 2018; Memel & Ryan, 2017; Troyer et al., 2011). These findings highlight unitization as a promising strategy that might help alleviate age-related deficits in associative recognition by allowing familiarity-based associative retrieval.

Nevertheless, for unitization to be a viable mnemonic strategy in real-life situations, several conditions should be met. First, as was described before, the associative-deficit hypothesis suggests that an area of particular difficulty amongst older adults is the formation of new associations between otherwise unrelated materials (Naveh-Benjamin, 2000). For example, if one placed their keys in an unusual location (e.g., on the microwave) the novel spatial relations between the keys and the microwave, which would allow them to find their keys later on, is harder to encode for older than younger adults. Therefore, to serve as a useful strategy, unitization should be applicable even when the relations between the to-be-encoded stimuli are novel or arbitrary. In our previous studies (e.g., Tibon, Gronau, et al., 2014), we have noted that unitization strategies can be viewed as driven by either top-down or bottom-up cognitive processes. Top-down approaches to unitization focus on encoding instructions to process multiple items as a single unit (in high-unitization conditions) or as separate elements of the same episode (in low-unitization conditions). Such instructions can take the form of compound definition versus use-in-sentence encoding of words (as will be used here; details below), or of encoding source and item information in an internal versus an external manner, e.g., “imagine each item in the colour indicated by the background screen colour” versus “imagine why the item would be associated with a stop sign or dollar bill” (Bastin et al., 2013; Diana et al., 2011). In contrast, bottom-up approaches are based on maximizing item features or associative information that might foster unitization. In this case, the instructions remain the same across all conditions, but inherent or presentation-related features of the stimuli are manipulated, to engender differential degrees of unitization (Tibon, Gronau, et al., 2014). For example, high-unitization stimulus pairs can differ from low-unitization pairs in their pre-existing semantic or schematic relationships (e.g., Han et al., 2023; Li et al., 2019; Tibon, Ben-Zvi, et al., 2014) or in their spatial configuration (e.g., plausible vs. implausible; Bridger et al., 2017; Huffer et al., 2022).

Most studies investigating the contribution of unitization to memory at older age employed bottom-up unitization strategies, relying on some pre-existing relations between encoded materials (e.g., Delhaye et al., 2018; Delhaye & Bastin, 2018; Memel & Ryan, 2017; Troyer et al., 2011; Zheng et al., 2015; but see Bastin et al., 2013). In the current study, we instead used a top-down unitization paradigm to ensure that this strategy is similarly useful when initially unrelated information is remembered. More specifically, we used the definition/sentence paradigm which is commonly used in the field in general, but less explored in the context of ageing. In this paradigm, participants are given an initially unrelated word pair such as CLOUD-LAWN in the context of either a definition (e.g., “a garden used for sky-gazing”) or a sentence (e.g., “the ______ could be seen from the ______”). The former, but not the latter, creates a new unit that allows the two words to be encoded as a compound. The first study to employ this procedure (Quamme et al., 2007) showed that amnestic patients with damage to the hippocampus and severe recollection deficits were nonetheless able to remember pairings of initially unrelated words presented in the context of a definition. In other words, although the patients struggled to remember non-unitized pairs, their memory for unitized pairs was relatively intact. Other studies employing this paradigm also supported the notion that recollection might not be necessary for the recognition of novel pairings, and that unitization can enable familiarity-based retrieval (e.g., Bader et al., 2010; Haskins et al., 2008; Kamp et al., 2016). In the current study, top-down unitization, manipulated as definition vs. sentence encoding, was used to attenuate the age-related associative deficit for initially unrelated materials.

In addition to the ability to utilize top-down unitization, a second condition that should be met for unitization to be a viable strategy, is for individuals to be able to initiate it as a strategy outside specific laboratory settings. In the paradigm described above, word pairs are accompanied by additional information (either a definition or a sentence), which provides the scaffolding for the mnemonic strategy that is being used. In everyday situations, however, such additional information is unlikely to be provided and instead needs to be generated by the individuals themselves. Therefore, in the current study, participants encoded the words in the context of a definition/sentence that was provided, but also in the context of a definition/sentence that they generated themselves. Whilst the current study used generation to establish unitization as a useful strategy in real-world situations, generation of memoranda in itself has been recognized as an important encoding strategy that can improve memory; known as the well-established generation effect where information is better remembered if it is actively created from one’s own mind rather than simply read (Jacoby, 1978; Slamecka & Graf, 1978). In lab settings, the basic generation paradigm involves the presentation of some type of paired-associates list to participants. Half the pairs are provided in their intact form (e.g., COLD, HOT), and the participants are instructed to read them. For the remaining items, participants are presented with the first half of the pair (COLD, _____) and are provided with a rule that they should use to generate the second half, such as the creation of synonyms, antonym, or rhymes. Later on, participants are asked to retrieve the target word (i.e., “HOT”), and often perform better when the target word was generated compared to when it was read (e.g., Bertsch et al., 2007). A large body of work shown that the generation effect is robust for the information that is generated across a variety of experimental procedures for both younger and older adults (e.g., McCurdy & Leshikar, 2022; McGillivray & Castel, 2010; for review see Bertsch et al., 2007).

With regard to the effect of ageing, studies have generally shown that the generation effect persists with normal ageing (e.g., Brown et al., 1993; McCurdy & Leshikar, 2022; McFarland et al., 1985; Pesta et al., 1996), although some variations between younger and older adults were observed. In particular, Taconnat & Isingrini, (2004) showed that the generation effect is comparable amongst younger and older adults when the task requires generation of semantic associates. However, for other generation tasks (rhymes, anagrams), for which processing is arguably shallower (see level of processing framework; Craik & Lockhart, 1972), the effect remains robust for younger adults but decreases (or even absent) for older ones. To account for their findings, the authors suggested that the generation effect can be driven by pre- and post-generation processes. Pre-generation processes are necessary for generation, automatic, and driven by the task. Adherently, according to the authors, in the semantic generation task which involves deep processing of the initial cue, semantic operations that are required for memorization occur prior to generation and are driven by the task. Post-generation processes, on the other hand, are self-initiated, controlled, and effortful. The rhyme-based generation task requires only one phonological process that does not constitute a very effective process for retaining information in memory and therefore relies on (additional) self-initiated processes for memorization. Accordingly, the authors suggested that age-effects on the generation effect reflect older adults’ reduced ability to spontaneously use processes that assist memorization, which is required to optimize encoding in shallow (e.g., rhyme-based) tasks, but not in deeper (e.g., semantic) tasks.

The effects of generation on associative information are less clear, however. It was suggested that generation tasks require that participants pay attention to the item itself, increasing item-specific encoding, but at the cost of the encoding of associations between the item and elements of the surrounding context (e.g., Jurica & Shimamura, 1999; McDaniel et al., 1988; Mulligan et al., 2006). Nevertheless, the alternative ‘associative strengthening’ view suggests that generation enhances recollection which entails recovery of context-specific information (e.g., Greenwald & Johnson, 1989; Marsh, 2006; Marsh et al., 2001; see McCurdy et al., 2020 for a recent Meta-analysis). It should be noted that in previous studies examining the generation effect, the items-to-be remembered (i.e., the targets) were self-generated. In contrast, in the current study, the targets were provided and the scaffolding binding information was generated instead. The influence of generation in this case was therefore hard to predict. Nevertheless, to utilize unitization as a viable strategy in real-life situations, it was important to establish that it can also occur when binding information is generated.

Following Bader et al. (2010) and Quamme et al. (2007), we operationalized “unitization” as the difference between two words being linked by a definition (e.g., CLOUD/LAWN, A garden used for sky-gazing), relative to two words being linked by a sentence (e.g., CLOUD/LAWN, The ______ could be seen from the ______). This manipulation of encoding instructions was done during an initial study phase. This scaffolding binding information (i.e., either a definition or a sentence) was either provided or generated by the participant. We hypothesized that, if top-down unitization can promote familiarity-based associative retrieval, then the associative deficit in older vs. younger adults will be smaller for definition vs. sentence encoding. Generation effects were also investigated, although we did not have specific predictions regarding their pattern. Importantly, we reasoned that a significant amelioration of associative deficits in the self-generation condition, would indicate that unitization is a viable mnemonic strategy.

## Methods

The methods used in this study carefully followed those that were registered with our Stage 1 report, available here: https://osf.io/n3kqd.

### Ethics information

This study was approved by the ethics committee of the School of Psychology, University of Nottingham (reference number: F1356R). Participants provided informed consent and were compensated for their time with £8 per hour.

### Participants

The sample size for the study was determined using a Bayesian “sequential design with maximal n” (Schönbrodt & Wagenmakers, 2018). Using this approach, the experiment was due to run in batches (with 32 participants in the first batch, and 16 participants in each additional batch), calculating the Bayes factor (BF) in favour of our key hypothesis (see below) after each batch. For the purpose of the simulations, we used a maximal n = 500 per group. Although this number exceeded our feasibility limit, it was chosen to allow us to fully appreciate the power of our design using various sample sizes. Once the simulation was conducted with the full sample size, the results were capped and interpreted for smaller sample sizes.

**Figure 1:**
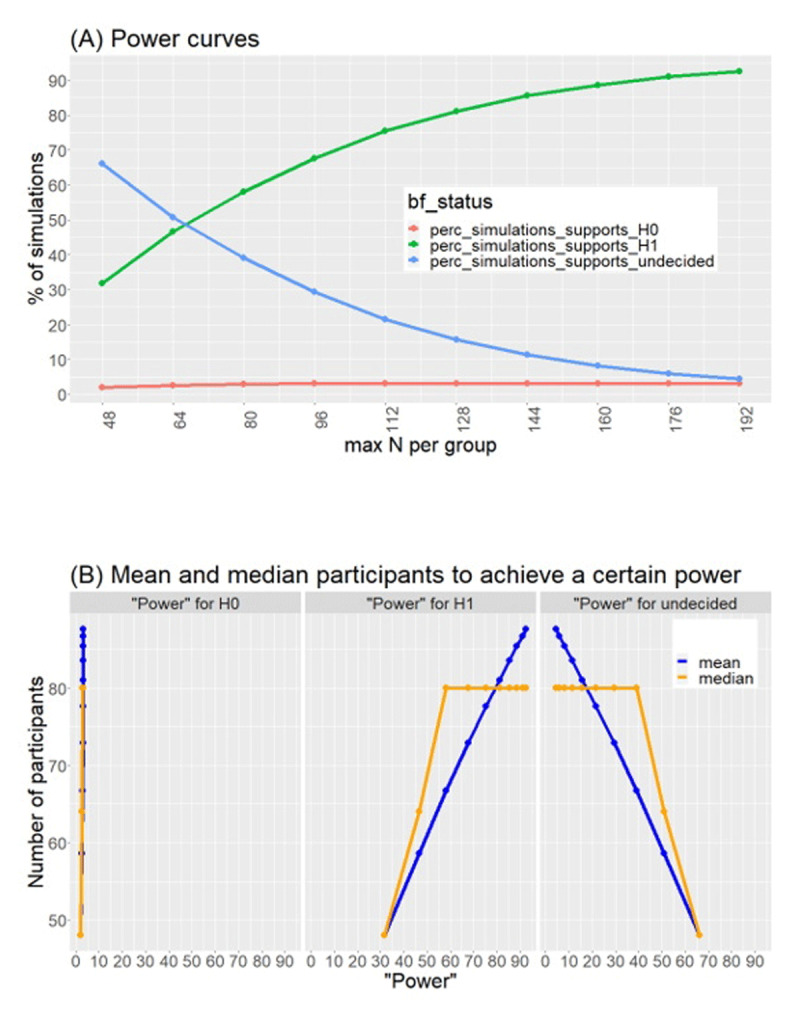
**Results of simulations for sample-size determination**. We simulated an effect size of 0.3 (medium-small). Panel A (top) shows power curves for % simulations that showed support for H0 (red), H1 (green) or were inconclusive (blue). Panel B (bottom) shows estimations of mean and median number of participants required to achieve a certain power.

We ran simulations (using 10,000 iterations) in order to determine the maximal number of participants that fall within our feasibility limits, but that would also be informative. The results of these are available on https://osf.io/n3kqd, and are summarised in Figure 1. For each iteration we generated a sample of n = 32 with two values per participant, where the differences between these values are drawn from a normal distribution, with a small-medium effect size (d = 0.3). This effect size was chosen based on two previous studies in which similar stimuli were used, and the design included the key effect designated to be used for the stopping criteria (namely, better memory for definition vs. sentence encoding) (Parks & Yonelinas, 2015; Tibon et al., 2018). In these studies, large effect sizes were obtained (Cohen’s d = 2.1 in Parks & Yonelinas, 2015 [exp 1]; Cohen’s d = .61 in Tibon et al, 2018 [exp 1 & 3]). Nevertheless, to avoid an overinflated estimation of effect size, we set d = 0.3 as a lower, more conservative value for our simulations.

The simulations were conducted using the cbu_bayesian_sequential_design toolbox (https://github.com/lbokeria/bayesian_sequential_design_simulations; Bokeria et al., 2022). We used a one-sided paired Bayesian t-test with a Cauchy prior scaled at sqrt(2)/2 (medium scaling) to calculate Bayes Factors for the simulated data. The stopping criteria for the iteration was set to BF_10_ > 6 or < 1/6. Thus, if the BF was greater than 6 (which would indicate support for our main hypothesis that the effect of × > 0, also called the “alternative hypothesis”, BF_10_) or lower than 1/6 (which would indicate support for the null hypothesis that effect of × = 0, BF_01_), the iteration ceased, and the next one was initiated. If, however, BF_10_ was < 6 and > 1/6, the iteration repeated with another batch of 16 (N = 48, N = 64, N = 80…), until a BF_10_ of 6 or 1/6 was obtained, or until the maximum number of participants had been reached.

The results of the simulations are shown in Figure 1. Panel A shows the percentage of simulations that reached the criteria (BF_10_ of 6 or 1/6) or were inconclusive for various maximal Ns. Note that because the true effect size in this simulation is 0.3, BF_10_ of 6 is considered a true positive result, whereas BF_10_ of 1/6 is a false negative. Panel B provides an estimation of power, by showing the mean number of participants required to reach the specified criteria (that is, to “exit” the iteration). As shown in the figure, with a maximal N of 160 per group, 90% of the simulations were ceased after reaching a BF_10_ of 6. Moreover, the average number of participants required to achieve a power of 90% was 90. We therefore set the maximal N for our study to be 160 per group (320 in total), with the expectation that ~90 participants per group (~180 in total) would be sufficient to reach our stopping criteria. Thus, given the above results, we set the stopping criteria to be (1) BF_10_ > 6 for the key analysis (see above); (2) BF_01_ > 6 for the key analysis; or (3) total N = 320.

Participants were recruited from Prolific (www.prolific.co), based on the following prescreening criteria: age range 18-35 for the Young Adults group and 65-85 for the Older Adult group, native English speakers, with normal or corrected-to-normal vision, and not diagnosed with dementia, mild cognitive impairment (MCI), attention deficit hyperactivity disorder, dyslexia, or any other developmental or learning disabilities. In accordance with our Stage 1 report, participants were excluded prior to analysis due technical problems (N = 2, one from each age group), reports of three or more complaints in the memory complaints questionnaire (N = 36 younger adults, N = 28 older adults), and failure to complete ‘catch’ trials (N = 10 older adults). Moreover, following data analysis, participants whose performance at the task were at or below chance level in two or more experimental conditions were excluded (N = 12 younger adults, N = 8 older adults). Finally, individual trials were excluded if the reaction time (RT) during retrieval was very fast (<300 ms) or very slow (>3 SD above the participant’s average RT). Excluded participants were replaced by others. As the Bayes factor for the key analysis (see above) has only reached the stopping criteria with the last batch, the final sample consisted of 160 younger (65 females and 95 males, mean age = 28.46 years, SD = 4.60) and 160 older participants (86 females and 74 males, with a mean age = 70.14 years, SD = 4.46).

### Ceiling/floor performance and task adjustment

Following a pilot study (see ‘Pilot data’ section below), we expected participants in both groups to reach accuracy rates above ~70%. Nevertheless, we took precautions to ensure that performance remains interpretable (i.e., not at floor or at ceiling) in the current study. Performance was assessed after data have been collected from the first 16 participants (8 from each group; one for each of the 16 stimulus lists). Floor performance was defined as a mean %correct responses < 50%, and ceiling performance was defined as a mean %correct responses > 95% across all conditions. In addition, we examined the results of the documented practice trials (see below) for the first 8 participants from each group, to ensure that participants were able to complete the task. Namely, we verified that within each group, most participants were able to generate adequate outputs for at least half of the practice trials in each generation block (i.e., 2/4 for that block).

### Materials

Stimuli were obtained from the dataset used in our previous study (Tibon et al., 2018), which is a modified version of the dataset used in Haskins et al. (2008). This stimulus dataset included 288 entries, each was comprised of semantically unrelated word pairs and their corresponding definition and sentence. For example, one entry in this dataset included the word-pair CLOUD LAWN, which corresponds to the definition “A garden used for sky-gazing” and to the sentence “The _____ could can be seen from the ______”. For the current study, 192 entries were used, and the stimulus set was divided into 16 sub-lists with 12 entries in each sub-list, to allow full counterbalancing across experimental conditions.

### Design and Procedure

**Figure 2:**
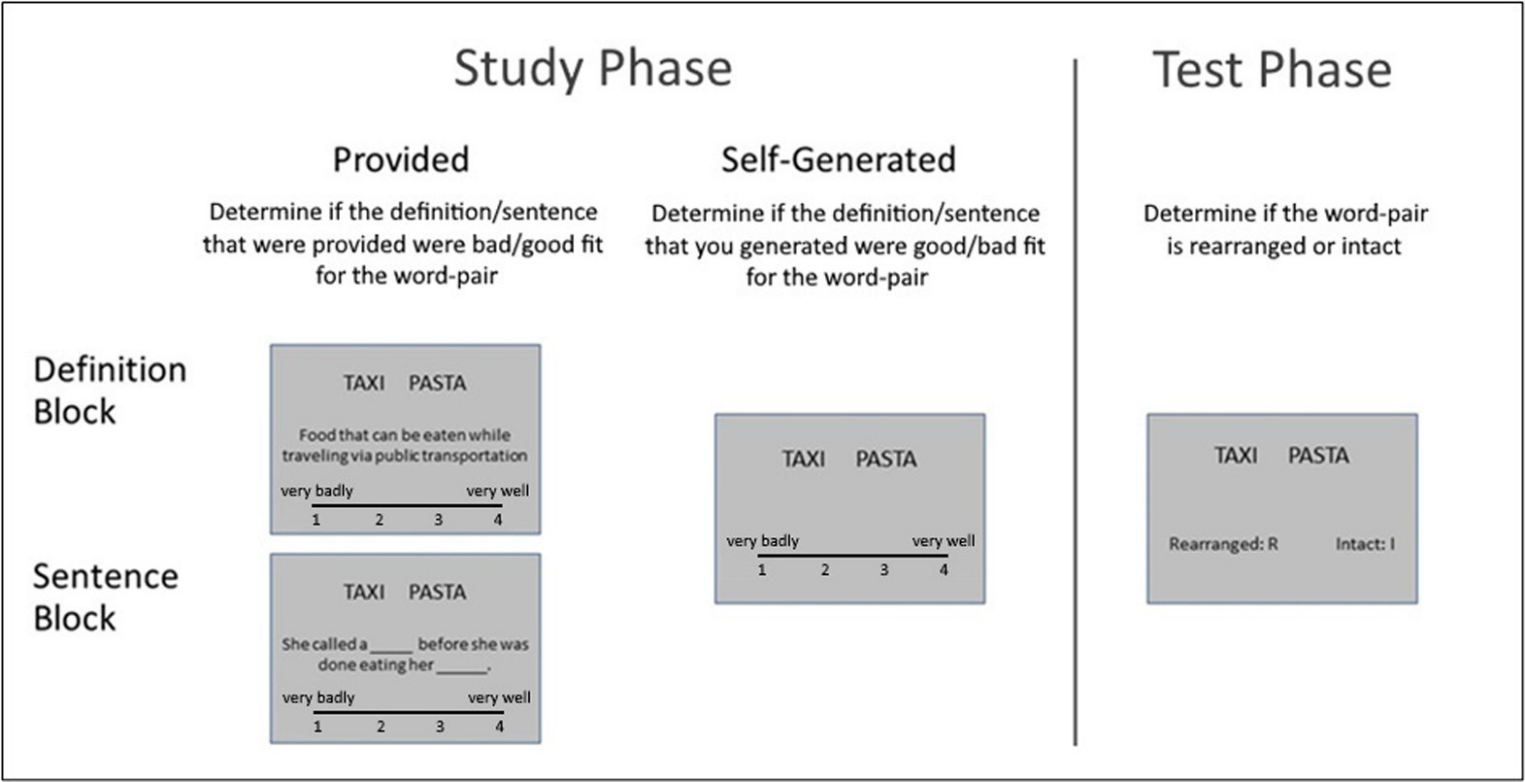
**Schematic depiction of the experimental design**. During the study phase, participants studied word-pairs in the context of definition or sentences that are either provided or self-generated. In a subsequent test phase, they discriminated between intact (studies) and rearranged pairs.

The design of the study is illustrated in Figure 2. This was an online study, and participants used their own personal computers to participate. They were recruited via Prolific (www.prolific.co) based on the eligibility criteria described above. An online information sheet was provided, followed by a consent form and the collection of demographic information (age and gender). Once consent was given, participants answered 5 yes/no questions, referring to whether they feel that they were: (1) forgetting where things were placed; (2) unable to recall the names of good friends; (3) unable to follow and recall conversation; (4) having memory problems; and (5) considering their own memory to be worse than others’ of a similar age. A previous study (Lam et al., 2005) showed robust relations between frequency of memory complaints in this questionnaire and scores on the Clinical Dementia Rating (CDR) scale; a clinical scale commonly used to assess the severity of dementia. Moreover, with a cutoff of 3 or more memory complaints, this memory questionnaire demonstrated a sensitivity of 70.4% in identifying early dementia.

The computerised task (developed with PsychoPy; Pierce et al., 2019; and administered via Pavlovia, www.pavlovia.org) then started. The design of the study included unitization condition (definition, sentence) as a within-subjects factor, generation condition (provided, generated) as a within-subjects factors, and age group (young, old) as a between-subjects factor. The dependent measure for the key analysis was the associative d’ scores, as detailed below (see ‘data analysis’ section).

The task was comprised of 4 study-test blocks (48 trials in each block), with each block including a different encoding condition: during the definition-provided (DefP) encoding condition, a word pair (e.g., CLOUD GARDEN) was presented, accompanied by a definition (e.g., “A garden used for sky-gazing”), and participants were instructed to rate the pair as a whole on a scale ranging from 1 (“very badly”) to 4 (“very well”) according to how well the definition combined the meanings of the two words into a sensible compound. In the sentence-provided (SenP) encoding condition a word pair accompanied by a sentence with two blanks appeared (e.g., “The _____ could be seen from the ______”), and participants were instructed to indicate whether the two words fit into the sentence frame on a scale ranging from 1 (“very badly”) to 4 (“very well”). In the definition-generated (DefG) and sentence-generated (SenG) conditions, only the word pair appeared, and participants were asked to generate their own definition/sentence and to indicate how well the words fit with these using the same scale as before.

Study trials began with a 500 ms fixation cross. Next, a word-pair and binding information (i.e., either a definition or a sentence) were displayed for 8,000 ms one above the other, slightly above and below central vision. Sentence frames were constructed with two blank spaces, where the first item was intended to fit into the first space, and the second item into the second space. Participants were asked to respond by pressing the “F” keyboard key to indicate good fit, or the “J” key to indicate bad fit. Only responses provided within the 8,000 ms time window, while the stimuli appeared on the screen, were recorded. Once the response was provided, a white dot appeared on the screen to indicate to the participant that their response had been recorded. The stimuli remained on the screen for the remaining time of the trial to equate exposure duration and total duration of encoding blocks across participants.

Each study block was followed by a filler task in which participants solved simple arithmetic problems. In this task, multiplication problems with two operands between 2 and 9 were presented horizontally at the centre of the screen for 300ms (e.g., 3 × 4). Participants pressed the space bar once they were able to provide an answer (being that during the presentation of the stimuli, or after they disappear from the screen) and then typed their answer and hit Enter to proceed to the next trial. We chose these specifications for the filler task (multiplication, operands, presentation duration) as it was previously shown that under these specific conditions, performance is comparable for younger and older adults (Allen et al., 1997), and therefore not expected to result in differential engagement/frustration for younger vs older participants. Within each block, participants engaged with the filler task for 2 min, regardless of how many problems they were able to solve during this time.

The filler task was followed by a test block. Test blocks were identical for all encoding conditions. In these test blocks, participants discriminated between intact word pairs comprised of two items that were studied together, and rearranged word pairs comprised of studied items in new combinations. Test trials began with a 500 ms fixation cross, followed by a presentation of the word pair. Participants were asked to press the “I” keyboard key to indicate that the pair was intact and the “R” key to indicate that it was rearranged. Responses were self-paced (up to a maximum limit of 6,000 ms) and triggered the presentation of the next trial.

All the instructions were given to the participants before the start of the experiment. Before each block, a brief reminder was provided, alongside a practice block, consisting of 12 trials. Out of these, 8 were study-test trials of that specific block. The remaining 4 trials were practice-catch trials. Namely, to ensure that participants understood the task, and in particular, that they were able to generate adequate outputs for generation blocks, in these catch trials participants were asked to type in the definition/sentence that they came up with (in generation blocks) or that was provided (in provided blocks). The structure of these trials was identical to that of other practice-study trials, but after participants provided their rating, another screen appeared in which they were asked to type in the definition/sentence that was provided/generated. Participants were informed that such trials, in which they need to record their responses, will also appear during the task. Adherently, 4 catch trials were included in each experimental block, to ensure that participants retained their engagement with the task throughout the experiment. During both practice trials and the main task, these catch trials were randomly intermixed with other study trials, such that participants did not know in advance for which trial they will need to document information. The word-pairs that were presented during catch trials were included in the test (or practice-test) phase but not analysed.

Following the completion of the task, participants were directed to a post-experiment questionnaire which consisted of questions on whether the participants were able to complete the study without interruptions and open-ended survey fields which will give participants the opportunity to report any issues they encountered whilst performing the study.

### Pilot Data

A pilot study, which included the 4 block types described above (DefP, DefG, SenP, SenG), was conducted to confirm the usability of the task and stimulus set that was used in the current study. Note that even though the principled paradigm had been used several times before (e.g., Bader et al., 2010; Haskins et al., 2008; Kamp et al., 2016; Parks & Yonelinas, 2015; Quamme et al., 2007; Shao et al., 2016; Tibon et al., 2018), to our knowledge, this is the first time that it was used in an online study and so it was essential to establish that the task can be used in these settings. The main purpose of the pilot was to ensure the technical validity of the task, as well as our ability to detect two key findings: (1) overall accuracy rates that exceed chance level in all conditions and in both groups; (2) an overall age effect such that accuracy rates for young adults are greater than for older adults.

A total of 35 participants took part in the pilot study. Out of these, 20 participants (10 males, 9 females, 1 other/prefer not to say) were younger adults (mean age 24.47 years, *sd* = 2.5, range = 20–28) and 15 (7 males, 5 females, 3 other/prefer not to say) were older adults (mean age 69.62 years, *sd* = 5.68, range = 65–85). Pilot data and analysis code used for the pilot are available on https://osf.io/n3kqd. Note that whilst block order was counterbalanced in this pilot, the assignment of stimuli into the various experimental conditions was not. Therefore, any examination of experimental conditions is potentially confounded by stimulus-specific effects and cannot be interpreted. Furthermore, due to the relatively small sample size, and lack of full counterbalancing, we were not necessarily expecting to find significant effects, or indeed robust evidence (using Bayes factors). Instead, we were looking for numerical trends that can provide a solid basis for the validity of the experimental paradigm. Nevertheless, we did conduct the relevant statistical tests, and as shown below, these provided further support for our conclusions.

First, mean accuracy rates in all experimental conditions and in both groups exceeded chance level, ranging between 77% and 88% for younger adults, and between 70% and 78% for older adults. This was confirmed by one-sample t-tests comparing accuracy rates in each condition and each group against chance level (50%), which yielded a minimal t-value of 14.16 (all p-values < .001). Second, overall accuracy rates were higher for younger than older adults (82% vs 74.4%). This effect was confirmed by an independent sample t-test, *t*(22.86) = 2.19, *p* = .019. Comparable analyses using Bayesian t-tests (implemented with the BayesFactor toolbox in R; Morey & Rouder, 2024) generated Bayes factors greater than 4, suggesting that our hypotheses (i.e., of accuracy levels greater than chance and greater overall accuracy for younger than older adults) were preferred over the null hypotheses. Thus, overall, the results of the pilot confirmed the utility of our study.

### Data Analysis

Analyses were conducted using the BayesFactor package (Morey & Rouder, 2024) as implemented within JASP (version 0.19; JASP Team, 2024). For the key analysis, associative d’ scores, representing an unbiased measure of memory, were extracted for each experimental condition by calculating the difference between the z-transformed hit rates (intact pair identified as intact) and false alarm rates (rearranged pairs identified as intact). A one-sided Bayesian paired t-test with a Cauchy prior scaled at sqrt(2)/2 (medium scaling) was performed to examine whether d’ scores were greater for definition vs. sentence encoding when binding information was provided. The aim of this analysis was to replicate previous findings and establish the basic unitization effect in the current study. Sequential sampling concluded when the basic unitization effect was established (*BF_10_* = 10.70), which occurred at the same batch in which the maximum number of participants was achieved (see ‘Participants’ section above). Additional analyses were conducted to examine how this basic effect was modulated by other factors of interest.

First, the same analyses that were applied to the pilot data (see ‘Pilot data’ subsection above) were performed on the experimental data to evaluate whether greater overall accuracy rates for younger than older adults, as well as above-chance accuracy rates for both groups and in all conditions were obtained. Next, a 3-way Bayesian ANOVA was conducted with encoding condition (definition, sentence) and generation condition (provided, generated) as within-subjects factors and with age group as a between-subjects factor. The results of this analysis allowed us to determine whether the well-documented unitization effect was modulated by age. We hypothesised that when binding information is provided, definition encoding can attenuate age-related decrease in associative memory. We therefore predicted an age × encoding condition interaction, such that both age groups would benefit from definition encoding when binding information is provided (d’ following definition encoding > d’ following sentence encoding), but this effect will be greater for older than younger adults. In addition, this analysis allowed us to investigate whether memory was better or worse when binding information is generated (rather than provided), whether definition encoding also benefits memory when the binding information is generated, and if this effect is further modulated by age. Additional exploratory analyses, detailed in the Results section, were performed to further investigate the effects that were obtained. Following our preregistration and the *Journal of Cognition* guidelines, we interpret Bayes Factors (BFs) greater than 6 as providing conclusive evidence.

### Reproducibility considerations

Raw data, processed data, task materials, code, pilot data, and approved Stage 1 protocol are available on https://osf.io/rx3sq/. All code used in the study were re-executed and verified by another researcher not directly involved in the project.

## Results

### Registered Analyses

Our first registered analysis aimed to examine whether (and when) our stopping criteria had been satisfied, such that data collection could be ceased. As described above, to determine the final sample size, a one-sided Bayesian t-test contrasting d’ scores for definition vs. sentence encoding when binding information is provided was conducted for the first batch, and again with each additional batch. With the final sample, this analysis revealed a Bayes factor of *BF_10_* = 10.70, indicating conclusive evidence for the unitization effect when binding information was provided. Note that as the BF for this analysis only exceeded 10 after the last batch was added, our predetermined maximal N of 320 participants was used in this study.

Following that, and to further verify the utility of the results, we ran additional registered analyses, aiming to ensure that accuracy exceeds chance level. Mean accuracy rates across all experimental conditions ranged between 75% and 79% for younger adults and between 72% and 79% for older adults. One-sample t-tests comparing accuracy rates in each condition and group against chance level of 50% showed that accuracy rates were significantly higher than chance, in all conditions and in both groups, with the minimal *t-*value of 24.90 (all *p*-values < .001). Corresponding Bayesian t-tests further corroborated these results, yielding Bayes factors greater than 1.7 × 10^53^. We then also compared accuracy rates between younger and older adults (77.54% vs. 76.14%, respectively), using an Independent Samples t-test with Welch correction, which did not reveal a significant difference between the groups, *t*(314.307) = 1.13, *p* = .26. A Bayesian Independent Samples t-test further provided moderate evidence for the null hypothesis of no difference, *BF_01_* = 4.39.

**Figure 3:**
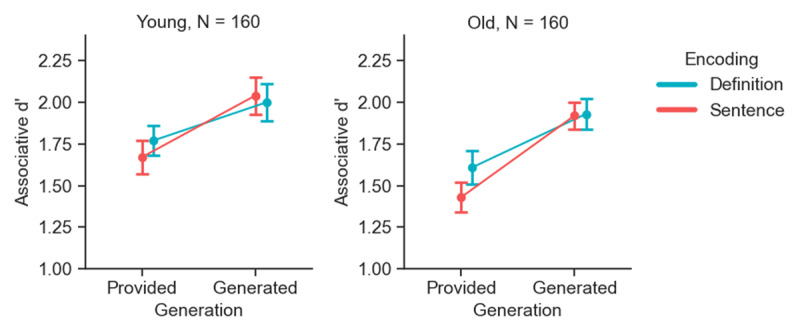
**Associative recognition memory performance across encoding and generation conditions by age group**. Associative d’ scores in the encoding and generation conditions, used in the main analysis, are shown. Separate plots depict data for young (left panel) and old (right panel) adults. Error bars represent 95% within-subjects confidence intervals.

These preliminary steps were followed by our key registered analysis, in which we assessed the effects of unitization, generation, age, and the interactions thereof, on associative recognition. A 3-way Bayesian ANOVA was conducted with encoding condition (definition, sentence) and generation condition (provided, generated) as within-subjects factors, age group (young, old) as a between-subjects factor, and associative d’ scores as the dependent variable. The results are depicted in Figure 3 and fully detailed in Table 1. This analysis provided conclusive evidence for the generation effect (*BF_incl_* = ∞) such that d’ scores in the generation condition were greater than in the provided condition. However, no conclusive evidence was found for encoding condition (*BF_excl_* = 1.96) or the interaction between encoding and generation (*BF_incl_* = 1.76). Similarly, no conclusive evidence was found for the main effect of age group (*BF_excl_* = 3.42), suggesting no conclusive differences in overall d’ scores between younger and older adults. Additionally, there was also no conclusive support for the interaction between age group and generation (*BF_excl_* = 3.60). Finally, the analysis revealed conclusive support for a null interaction between encoding condition and age group (*BF_excl_* = 11.04), and the three-way interaction of encoding × generation × age group (*BF_excl_* = 63.11).

**Table 1:** **Bayes Factors for the main analysis**. *BF_incl_ and BF_excl_ denote the Bayes Factors in favour of including and excluding the effect, respectively*. We interpret Bayes Factors (BFs) greater than 6 as providing conclusive evidence. *∞* indicates a Bayes Factor so large it exceeds the software’s numerical limits, reflecting overwhelming evidence for the model.


EFFECTS	BF_INCL_	BF_EXCL_

Encoding	0.51	1.96

Generation	∞	

Encoding × Generation	1.76	0.57

Age group	0.29	3.42

Encoding × Age group	0.09	11.04

Generation × Age group	0.28	3.60

Encoding × Generation × Age group	0.02	63.11


### Exploratory analyses

#### Further examination of Unitization effects

The results of the registered Bayesian 3-way ANOVA described above did not support the presence of a unitization effect, i.e., memory was not conclusively enhanced when definition (vs. sentence) encoding was employed. Nevertheless, as also mentioned above, conclusive evidence for the unitization effect were obtained in the current study when binding information was provided (i.e., *BF* > 10 for the analysis that determined when data collection was to be ceased). This suggests that the lack of conclusive evidence in favour of an overall effect might stem from a null unitization effect of encoding condition when binding information is generated (rather than provided). To formally test this, we used a one-sided Bayesian paired-sample t-test to contrast associative d’ scores for definition vs. sentence encoding within the generation condition. This showed robust support for the null hypothesis of no difference (generated *BF_01_* = 20.578), suggesting that contrary to when binding information was provided, in the current study, unitization did not improve memory when binding information was generated.

As for when binding information was provided, we have already shown above that in this case a unitization effect is obtained. However, to further understand the nature of this effect, we used independent sample one-sided Bayesian t-tests within the provided condition, to compare d’ scores for younger vs. older adults in sentence encoding and definition encoding. This showed that for sentence encoding the alternative hypothesis of higher d’ scores for younger than older adults was preferred (*BF_10_* = 4.18). In contrast, for definition encoding, the null hypothesis of no difference between the groups was preferred (*BF_10_* = 0.65). Notably, however, as discussed below, neither of these yielded conclusive support.

#### Bayesian Model Comparison

To complement the Bayesian model averaging approach in our registered analysis, we performed Bayesian model comparison to evaluate the relative evidence across all plausible models. The results revealed that the model including the generation factor alone had the highest Bayes Factor (*BF_m_* = 5.64), suggesting that models incorporating this factor are more likely than those without it. However, the Bayes Factor did not exceed the predefined threshold for strong evidence (*BF* > 6), rendering this effect suggestive rather than conclusive. The second-best model, which included the factors of encoding, generation, and their interaction (encoding × generation), yielded a very similar *BF_m_* = 5.59, indicating that these two models were closely matched in their explanatory power (with *BF_10_* = 0.99 for the second model relative to the first). Other models exhibited *BF_m_* values below 3, indicating weaker support.

Given that the two top-performing models were closely matched in evidence, and that Bayes factor estimates in Bayesian ANOVA are subject to variability due to Monte Carlo sampling (Pfister, 2021), we assessed the stability of these findings by repeating the model comparison across multiple iterations. The results shown in Table 2 illustrate that in 3 out of 10 iterations, the generation-only model performed better than all other models, while in 7 out of 10 iterations, the model that included encoding, generation, and their interaction (encoding × generation) was the best model. This variability highlights the stochastic nature of the Bayesian sampling process, sometimes resulting in the model that included generation, encoding and the corresponding interaction effect (encoding × generation) emerging as more prominent. Nonetheless, the overall conclusions demonstrate the robustness of the generation effect while acknowledging the occasional prominence of an encoding effect and an interaction effect.

**Table 2:** **Bayesian Model Comparison across ten iterations**. *BF_m_* denotes the Bayes Factor in favour of the model relative to the null model. Bolded *BF_m_* values indicate evidence supporting the better-performing model.


ITERATION	GENERATION (BF_M_)	ENCODING + GENERATION + ENCODING × GENERATION (BF_M_)

1	**5.63**	5.58

2	**5.98**	5.69

3	4.75	**8.05**

4	5.59	**5.86**

5	5.42	**5.92**

6	5.16	**5.88**

7	5.44	**5.71**

8	**5.39**	5.31

9	5.60	**5.74**

10	4.67	**8.02**


#### Median and tertile split of participants based on overall memory performance

Grounded in previous research showing dissociable utilization of mnemonic mechanisms in high and low memory performers (e.g., Bridger and Mecklinger, 2012), these exploratory analyses aimed to investigate whether participants’ overall memory performance interacted with their outcomes in the various experimental conditions. To achieve this, participants within each age group (old and young) were categorized into performance bins using two different splits, indexed by overall associative d’ scores across associative memory tests in all conditions. First, we used median split to divide participants into two groups—high and low performers—based on the median overall associative d’ score of their respective age group. Second, we used tertile split to divide participants into three groups—low, medium, and high performers—based on tertiles d’ within each age group. We note that as the choice between median and tertile splits can be somewhat arbitrary; both are reported to ensure transparency.

These performance bins were then included as an additional between-subjects factor in separate four-way Bayesian ANOVAs, alongside encoding, generation, and age group as main factors. To reduce the high computational demands, posed by the large number of models produced by these analyses, we used Laplace approximation (Rue et al., 2009) as the integration method for the models, and reduced the number of posterior samples from 1,000 to 500. While this was done to allow us to investigate the potential effects of performance, we acknowledge that these steps reduce model accuracy / stability and therefore results should be treated with caution.

**Figure 4:**
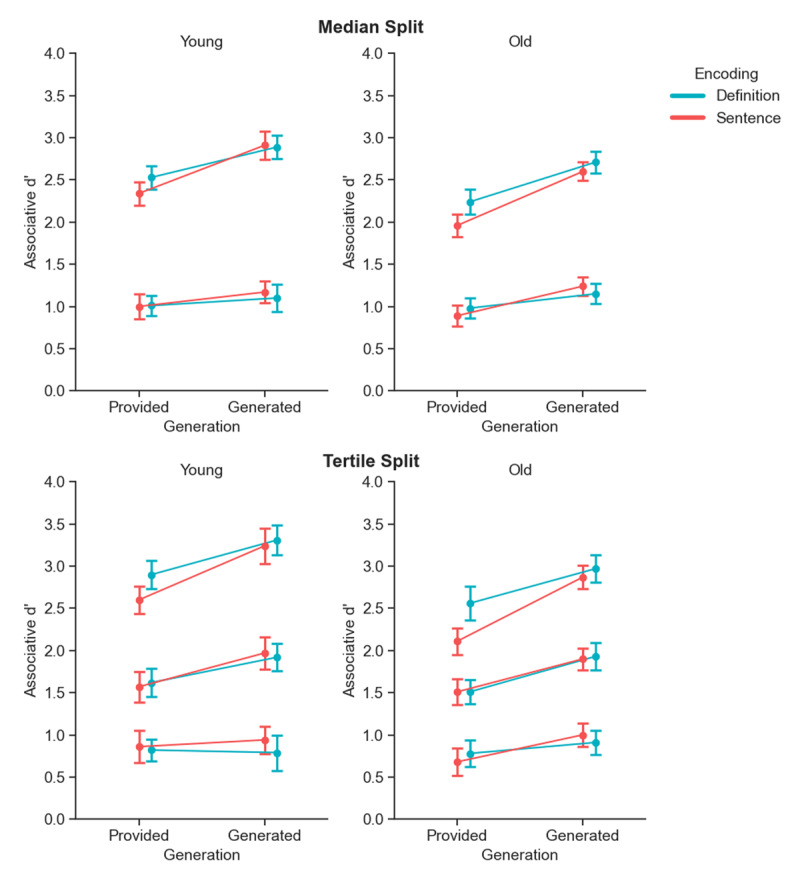
**Median and tertile split of associative recognition memory performance**. Associative d’ scores in the various encoding and generation conditions, for younger (left) and older (right) adults, categorized by participants’ performance. Performance is divided into two bins (median split, top row) and three bins (tertile split, bottom row) based on overall associative d’ scores. Error bars represent 95% within-subjects confidence intervals.

The results of these analyses are shown in Figure 4 and are further detailed in Table 3. Both the median and tertile splits provide conclusive evidence for a generation × performance bin interaction (BF_10_ = 922.03 for median split, and 2202.50 for tertile split). Post hoc comparisons showed that the generation effect was conclusive in the medium (*BF_10_* = 2.67 × 10^5^) and high (*BF_10_* = 2.88 × 10^11^) performance groups but not in the low-performance group (*BF_10_* = 1.94) indicating a generation effect that is more pronounced in participants who exhibit higher overall memory performance.

**Table 3:** **Bayes Factors for Four-Way ANOVA Across Median and Tertile Splits**. *BF_incl_ and BF_excl_ denote the Bayes Factors in favour of including and excluding the effect, respectively. ∞* indicates a Bayes Factor so large it exceeds the software’s numerical limits, reflecting overwhelming evidence for the model.


EFFECTS	MEDIAN SPLIT		TERTILE SPLIT	

	*BF_INCL_*	*BF_EXCL_*	*BF_INCL_*	*BF_EXCL_*

Encoding	0.38	2.62	1.17	

Generation	∞		∞	

Encoding × Generation	1.33		2.40	

Age group	2.62		954.54	

Encoding × Age group	0.11	8.77	0.15	6.82

Generation × Age group	0.34	2.99	0.35	2.83

Encoding × Generation × Age group	0.02	56.18	0.02	44.43

Performance bins	∞		∞	

Encoding × Performance bins	0.64	1.57	2.98	

Generation × Performance bins	922.03		2202.50	

Encoding × Generation × Performance bins	0.27	3.66	0.55	1.83

Age group × Performance bins	2.73		87.17	

Encoding × Age group × Performance bins	0.05	20.91	0.04	23.10

Generation × Age group × Performance bins	0.19	5.15	0.14	7.17

Encoding × Generation × Age group × Performance bins	7.376 × 10^–5^	13558	1.658 × 10^–5^	60323


Furthermore, a robust age group effect was revealed when participants were categorized using tertile splits, with strong evidence supporting a significant difference in recognition memory performance between older and younger adults (*BF_10_* = 954.54). In contrast, the effect of age group was inconclusive when a median split was applied (*BF_10_* = 2.62). Moreover, a conclusive age group × performance bin interaction was observed with the tertile split (*BF_10_* = 87.17), such that the difference in scores between younger and older adults varied across performance bins. Namely, as was indicated by one-sided Bayesian t-tests of overall associative d’, performed separately within each performance bin, higher scores for young vs. older adults were conclusively obtained for the high-performance group (*BF_10_* = 48550.22), but not in the medium (*BF_01_* = 2.22) or low-performance groups (*BF_01_* = 4.48). These findings suggest that the granularity of the performance binning method substantially impacts the detection of age-related differences in recognition memory performance.

Additionally, given the central role of establishing the basic unitization effect for older adults, we conducted post-hoc Bayesian t-tests within each tertile of older participants, focusing on the provided condition. Results indicated strong evidence for a unitization advantage only in the highest-performing tertile (*BF_10_* = 67.40). In contrast, the null hypothesis was preferred for both the lowest-performing group (*BF_01_* = 3.12) and the medium-performing group (*BF_01_* = 6.46). These findings suggest that, within our sample, the observed benefit from top-down unitization in the provided condition for older adults was driven by the tertile with higher associative memory performance.

#### Relations between age and subjective memory complaints

The current study aimed to examine how mnemonic strategies can be used to reduce age-related memory decline associated with healthy ageing. To this end, we excluded participants who might have MCI or dementia, based on their subjective reports. Nevertheless, to ensure that there are no potential residual effects of unhealthy cognitive ageing, we further examined the relations between age and subjective memory complaints (based on the 5 yes/no questions described, see Design and Procedure), to ensure that these are equated across the two age groups. A greater proportion of older adults reported a higher number of memory difficulties compared to younger adults (Table 4). However, Bayesian Contingency Table Tests provided inconclusive evidence (*BF_10_* = 1.19), suggesting that the two groups may not differ substantially in their self-reported memory difficulties.

**Table 4:** Self-Reported Memory Difficulties by Age Group.


AGE GROUP	MEMORY DIFFICULTIES			

	0	1	2	TOTAL

Young	121	25	14	160

Old	97	41	22	160

Total	218	66	36	320


*Note*. Each cell displays the observed counts.

## Discussion

The aim of the current study was to examine whether unitization can serve as a mnemonic strategy in real-life situations and to alleviate age-related memory decline. To this end, we argued, unitization should operate as a top-down self-initiated strategy. In support of this notion, the top-down unitization strategy employed in our study resulted in better associative recognition, reflected in greater d’ scores relative to a non-unitization condition. This suggests that unitization can indeed operate as a top-down strategy. However, this effect was limited to conditions in which unitizing information was provided, and thus in the current case, unitization did not operate as a self-initiated strategy. Taken together, these results imply that whilst unitization does have the potential to advance memory in real-life situations, further research is needed to optimize the conditions under which this can be achieved.

Using a variety of top-down tasks, previous research has shown that, under specific conditions, single-trial learning of arbitrary associations is possible via the unitization of two unrelated items. These studies generally find better memory for associative information that had been unitized compared to non-unitized associations, primarily using recognition tasks (Diana et al., 2008, 2011; Haskins et al., 2008; Quamme et al., 2007; Tu & Diana, 2021). Previous research had also shown that the advantage of unitization encoding persists (or sometimes elevated) in older adults even in the presence of an age-related memory decline (Ahmad et al., 2015; Bridger et al., 2017; Delhaye et al., 2018; Delhaye & Bastin, 2018; Huffer et al., 2022; Liu et al., 2024; Memel & Ryan, 2017; Zheng et al., 2015). However, these studies show an advantage when unitization relies on preexisting knowledge that supports the creation of episodic relations between item pairs (i.e., bottom-up unitization). Here we show that older adults can also benefit from top-down unitization; an important step in outlining the theoretical characteristics of unitization and in establishing it as a useful strategy in real-life situations. Moreover, to our knowledge, this is the first online (rather than lab-based) study that shows the advantage of top-down unitization, further demonstrating the feasibility of unitization to serve as a viable strategy that can be trained and used outside the lab.

Although unitization was beneficial when binding information was provided, it did not operate as a self-initiated strategy in the current study. Nevertheless, our results do provide conclusive evidence to the notion that self-generated binding information results in better overall associative recognition, relative to when binding information is provided. This was evident both in a registered analysis, showing robust evidence for the generation effect, as well as in an exploratory analysis in which various models were compared, and those that included the generation effect consistently fitted the data better than those that did not include this effect. As noted above, in previous studies the items-to-be remembered (i.e., the targets) have been generated and memory for these (vs. non-generated targets) was assessed. In contrast, in the current study, the targets were provided and scaffolding binding information was generated instead. The robust generation effect that was obtained suggests that, even by itself (i.e., regardless of any additional effects of unitization), self-generation of scaffolding information can serve as a strategy that benefits associative recognition. We suggest that this advantage can be viewed in terms of levels of processing (Craik & Lockhart, 1972). Namely, although semantic (deep) processing is required in both cases, when information is self-generated (vs. provided), the task requires further engagement that might further deepen the processing. Notably, an additional exploratory analysis, in which participants were divided into high- and low-performance groups, showed that while the generation effect was apparent in both groups, it was more pronounced in participants who performed better overall. This implies that while the generation of scaffolding information can benefit associative recognition in general, some additional steps should be taken to ensure that the effect is augmented for those who need it the most – those whose memory performance is relatively poor. To this end, the quality of the generated scaffolding information should be further investigated. It is possible that the nature of the generated information is different across high and low performers, age groups (e.g., more elaborative in older adults, thereby minimizing age-related differences), or more similar across encoding conditions (thereby hindering unitization effects). Due to practical constraints (namely that the task was already considerably long for an online task), we were not able to fully capture the information generated by participants. These issues can therefore be addressed in future studies in which specific conditions are targeted (e.g., generation only), to reduce time on task, and participants’ responses are fully documented.

The notion that unitization can serve as a mnemonic strategy particularly beneficial to alleviate age-related memory decline inherently assumes better memory performance in younger than older adults. However, the current study did not show evidence for that. Surprisingly, although numerically d’ scores were slightly higher for younger than older adults, this difference was not conclusive, and in fact, the null hypothesis of no difference was (inconclusively) preferred over the alternative hypothesis (*BF_10_* < 1). The only analysis in which clear age effects were observed was when participants were divided into 3 bins based on their task performance. Under these conditions, a robust effect of age group was obtained, which further interacted with the performance group such that for the best-performing group (but not for other groups), memory was better for younger than older adults. Note that robust evidence for this pattern was obtained when a tertile split was used, but not for a median split, suggesting that task performance for younger and older adults is similar in the majority of the sample. As noted by Greene & Naveh-Benjamin (2022), it is plausible that this unexpected absence of age effect is due to specific characteristics of the current study, and in particular, the online administration of the task. For example, it could be that the older adults who are able to engage and complete a relatively complex computerised online task are those who function very well to begin with and experience reduced cognitive decline relative to their age. Therefore, their memory performance might be more similar to the group of young adults than that of the “average” older adult. Indeed, an exploration of the distribution of subjective memory complaints amongst younger and older adults in the current sample suggested that even though older adults had more memory complaints than younger adults, and that the alternative hypothesis of dependence between age-group and number of complaints was preferred over the null, the evidence for this were inconclusive. This suggests that the two groups are relatively similar, both in terms of their objective performance in the task and their subjective evaluation of their own memory—potentially more similar than in the typical population.

Another assumption that underlines the potential benefits of unitization in alleviating age-related memory decline, is that associative memory in older adults will be worse than in younger adults when non-unitized binding is achieved (i.e., via sentence encoding), but will not be affected, or will be affected to a lesser extent, when unitization was used (via definition encoding). This assertion rests on known characteristics of age-related changes in the neural mechanisms assumed to support unitization. In particular, it had been suggested that processing of unitized associations can be achieved via distinct neural mechanisms. Namely, instead of recollection-based hippocampal processing which tends to decline with age (Old & Naveh-Benjamin, 2008; Yonelinas et al., 2007), unitized associations can rely on item- and familiarity-based PrC processing (Dennis et al., 2024; Diana et al., 2007). Whilst this pattern did not emerge overall across the various generation conditions, it did emerge, to some extent, when binding information was provided. Specifically, whereas for sentence encoding the alternative hypothesis was preferred and evidence of better performance in young vs. older adults was obtained, for definition encoding the null hypothesis of no difference was preferred. However, the results of these comparisons, as well as those of the age-group × encoding condition interaction, did not surpass our predefined threshold (for all of these, BFs were lower than 6), and should therefore be treated as trends, and with caution.

One notable feature of our exploratory findings is the substantial heterogeneity in memory performance within each age group. By sampling through an online participant recruitment platform, we gathered a relatively large sample size and demographically diverse cohort and captured a wider range of associative memory abilities than may be observed in in-person laboratory studies. This variability may contribute to the reason the preregistered analysis revealed no conclusive main effects of encoding condition, age group, or encoding × generation interaction. Importantly, these effects only became apparent once performance ability was accounted for in our exploratory analyses. Specifically, the predicted patterns of encoding and generation effects were most evident in the highest-performing tertile of participants. This suggests that the observed effects were largely driven by a subset of individuals with stronger associative memory. Moreover, conclusive evidence for establishing the unitization effect only emerged when the final batch of participants was included. We speculate that the memory performance heterogeneity sampled, provides a more ecologically valid representation of the population. Thus, despite our suggestion above, that online administration potentially reduces age-related differences, cognitive abilities amongst older adults in our sample are still more diverse than in typical university-based samples. Consequently, efforts to translate findings from laboratory-based studies into real-world cognitive strategies should account for the broader variability in memory abilities found across the general population, rather than relying solely on effects observed in high-performing or narrowly sampled cohorts.

Overall, the results of the current study suggest that additional steps should be taken before unitization can be used as a mnemonic strategy that facilitates associative memory in real-life situations. In particular, more research is needed to examine and establish the conditions under which it can be operable with self-generated binding information. Without this, the ability of unitization to support independent new learning remains highly limited. To conclude, the current study adds to previous literature by further characterizing the conditions under which unitization can be used as a viable mnemonic strategy and highlights further steps that should be taken before this strategy can be used effectively beyond lab settings and in older adults. It may therefore inform the development of evidence-based cognitive strategies that could help attenuate age-related memory decline.

## Data Accessibility Statement

Raw data, processed data, task materials, code, pilot data, and approved Stage 1 protocol are available on https://osf.io/rx3sq/. All code used in the study were re-executed and verified by another researcher not directly involved in the project.

## References

[1] Ahmad, F. N., Fernandes, M., & Hockley, W. E. (2015). Improving associative memory in older adults with unitization. Aging, Neuropsychology, and Cognition, 22(4), 452–472. 10.1080/13825585.2014.980216 25396267

[2] Allen, P. A., Smith, A. F., Jerge, K. A., & Vires-Collins, H. (1997). Age differences in mental multiplication: Evidence for peripheral but not central decrements. The Journals of Gerontology. Series B, Psychological Sciences and Social Sciences, 52(2), P81–90. 10.1093/geronb/52B.2.P81 9060983

[3] Bader, R., Mecklinger, A., Hoppstädter, M., & Meyer, P. (2010). Recognition memory for one-trial-unitized word pairs: Evidence from event-related potentials. NeuroImage, 50(2), Article 2. 10.1016/j.neuroimage.2009.12.100 20045471

[4] Bastin, C., Diana, R. A., Simon, J., Collette, F., Yonelinas, A. P., & Salmon, E. (2013). Associative memory in aging: The effect of unitization on source memory. Psychology and Aging, 28(1), Article 1. 10.1037/a0031566 PMC3760335 23527745

[5] Beard, J. R., Officer, A. M., & Cassels, A. K. (2016). The World Report on Ageing and Health. The Gerontologist, 56(Suppl_2), Article Suppl_2. 10.1093/geront/gnw037 26994257

[6] Bender, A. R., Naveh-Benjamin, M., & Raz, N. (2010). Associative deficit in recognition memory in a lifespan sample of healthy adults. Psychology and Aging, 25, 940–948. 10.1037/a0020595 20822256 PMC3011045

[7] Bertsch, S., Pesta, B. J., Wiscott, R., & McDaniel, M. A. (2007). The generation effect: A meta-analytic review. Memory & Cognition, 35(2), 201–210. 10.3758/BF03193441 17645161

[8] Bridger, E. K., Kursawe, A.-L., Bader, R., Tibon, R., Gronau, N., Levy, D. A., & Mecklinger, A. (2017). Age effects on associative memory for novel picture pairings. Brain Research, 1664, 102–115. 10.1016/j.brainres.2017.03.031 28377157

[9] Bridger, E. K., & Mecklinger, A. (2012). Electrophysiologically Dissociating Episodic Preretrieval Processing. Journal of Cognitive Neuroscience, 24(6), 1476–1491. 10.1162/jocn_a_00152 21981675

[10] Brown, J. C., Niinikoski, J., & Duke, L. W. (1993). Generation effect and frequency judgment in young and elderly adults. Experimental Aging Research, 19(2), 147–164. 10.1080/03610739308253928 8319733

[11] Brubaker, M. S., & Naveh-Benjamin, M. (2014). The effects of presentation rate and retention interval on memory for items and associations in younger adults: A simulation of older adults’ associative memory deficit. Aging, Neuropsychology, and Cognition, 21(1), 1–26. 10.1080/13825585.2013.772558 23509862

[12] Craik, F. I. M., & Lockhart, R. S. (1972). Levels of processing: A framework for memory research. Journal of Verbal Learning and Verbal Behavior, 11(6), Article 6. 10.1016/S0022-5371(72)80001-X

[13] Davidson, P. S. R., & Glisky, E. L. (2002). Neuropsychological correlates of recollection and familiarity in normal aging. Cognitive, Affective, & Behavioral Neuroscience, 2(2), 174–186. 10.3758/CABN.2.2.174 12455684

[14] Delhaye, E., & Bastin, C. (2018). The impact of aging on associative memory for preexisting unitized associations. Aging, Neuropsychology, and Cognition, 25(1), 70–98. 10.1080/13825585.2016.1263725 27934542

[15] Delhaye, E., Tibon, R., Gronau, N., Levy, D. A., & Bastin, C. (2018). Misrecollection prevents older adults from benefitting from semantic relatedness of the memoranda in associative memory. Aging, Neuropsychology, and Cognition, 25(5), 634–654. 10.1080/13825585.2017.1358351 PMC6597361 28756745

[16] Dennis, N. A., Carpenter, C. M., & Becker, A. (2024). Examining the neural basis of unitization: A review. Cognitive, Affective, & Behavioral Neuroscience, 24(3), 389–401. 10.3758/s13415-024-01170-3 PMC11542030 38413465

[17] Diana, R. A., Van den Boom, W., Yonelinas, A. P., & Ranganath, C. (2011). ERP correlates of source memory: Unitized source information increases familiarity-based retrieval. Brain Research, 1367, 278–286. 10.1016/j.brainres.2010.10.030 20965154 PMC3095515

[18] Diana, R. A., Yonelinas, A. P., & Ranganath, C. (2007). Imaging recollection and familiarity in the medial temporal lobe: A three-component model. Trends in Cognitive Sciences, 11(9), Article 9. 10.1016/j.tics.2007.08.001 17707683

[19] Diana, R. A., Yonelinas, A. P., & Ranganath, C. (2008). The effects of unitization on familiarity-based source memory: Testing a behavioral prediction derived from neuroimaging data. Journal of Experimental Psychology. Learning, Memory, and Cognition, 34(4), Article 4. 10.1037/0278-7393.34.4.730 PMC2605011 18605864

[20] Donaldson, D. I., & Rugg, M. D. (1998). Recognition memory for new associations: Electrophysiological evidence for the role of recollection. Neuropsychologia, 36(5), Article 5. 10.1016/S0028-3932(97)00143-7 9699947

[21] Friedman, D. (2013). The Cognitive Aging of Episodic Memory: A View Based on the Event-Related Brain Potential. Frontiers in Behavioral Neuroscience, 7. https://www.frontiersin.org/articles/10.3389/fnbeh.2013.00111. 10.3389/fnbeh.2013.00111 PMC3752587 23986668

[22] Gabrieli, J. D. E. (1998). Cognitive neuroscience of human memory. Annual Review of Psychology, 49, 87–115. 10.1146/annurev.psych.49.1.87 9496622

[23] Graf, P., & Schacter, D. L. (1989). Unitization and grouping mediate dissociations in memory for new associations. Journal of Experimental Psychology: Learning, Memory, and Cognition, 15(5), Article 5. 10.1037//0278-7393.15.5.930 2522139

[24] Greene, N. R., & Naveh-Benjamin, M. (2022). Online experimentation and sampling in cognitive aging research. Psychology and Aging, 37(1), 72–83. 10.1037/pag0000655 35113615

[25] Greenwald, A. G., & Johnson, M. M. S. (1989). The generation effect extended: Memory enhancement for generation cues. Memory & Cognition, 17(6), 673–681. 10.3758/BF03202628 2811664

[26] Han, M., Li, B., Guo, C., & Tibon, R. (2023). Effects of emotion and semantic relatedness on recognition memory: Behavioral and electrophysiological evidence. Psychophysiology, 60(1), e14152. 10.1111/psyp.14152 35867964 PMC10078278

[27] Haskins, A. L., Yonelinas, A. P., Quamme, J. R., & Ranganath, C. (2008). Perirhinal cortex supports encoding and familiarity-based recognition of novel associations. Neuron, 59(4), Article 4. 10.1016/j.neuron.2008.07.035 18760692

[28] Hockley, W. E., & Consoli, A. (1999). Familiarity and recollection in item and associative recognition. Memory & Cognition, 27(4), Article 4. 10.3758/BF03211559 10479824

[29] Huffer, V., Bader, R., & Mecklinger, A. (2022). Can the elderly take the action? – The influence of unitization induced by action relationships on the associative memory deficit. Neurobiology of Learning and Memory, 194, 107655. 10.1016/j.nlm.2022.107655 35788058

[30] Jacoby, L. L. (1978). On interpreting the effects of repetition: Solving a problem versus remembering a solution. Journal of Verbal Learning and Verbal Behavior, 17(6), 649–667. 10.1016/S0022-5371(78)90393-6

[31] Jäger, T., & Mecklinger, A. (2009). Familiarity supports associative recognition memory for face stimuli that can be unitised: Evidence from receiver operating characteristics. European Journal of Cognitive Psychology, 21(1), 35–60. 10.1080/09541440802003140

[32] Jäger, T., Mecklinger, A., & Kipp, K. H. (2006). Intra- and inter-item associations doubly dissociate the electrophysiological correlates of familiarity and recollection. Neuron, 52(3), Article 3. 10.1016/j.neuron.2006.09.013 17088218

[33] Jetten, J., Haslam, C., Pugliese, C., Tonks, J., & Haslam, S. A. (2010). Declining autobiographical memory and the loss of identity: Effects on well-being. Journal of Clinical and Experimental Neuropsychology, 32(4), 408–416. 10.1080/13803390903140603 19787523

[34] Jurica, P. J., & Shimamura, A. P. (1999). Monitoring item and source information: Evidence for a negative generation effect in source memory. Memory & Cognition, 27(4), 648–656. 10.3758/BF03211558 10479823

[35] Kamp, S.-M., Bader, R., & Mecklinger, A. (2016). The Effect of Unitizing Word Pairs on Recollection Versus Familiarity-Based Retrieval—Further Evidence From ERPs. Advances in Cognitive Psychology, 12(4), 169–178. 10.5709/acp-0196-2 28154613 PMC5279856

[36] Koen, J. D., & Yonelinas, A. P. (2016). Recollection, not familiarity, decreases in healthy ageing: Converging evidence from four estimation methods. Memory, 24(1), 75–88. 10.1080/09658211.2014.985590 25485974 PMC4459941

[37] Lam, L. C. W., Lui, V. W. C., Tam, C. W. C., & Chiu, H. F. K. (2005). Subjective memory complaints in Chinese subjects with mild cognitive impairment and early Alzheimer’s disease. International Journal of Geriatric Psychiatry, 20(9), 876–882. 10.1002/gps.1370 16116581

[38] Li, B., Han, M., Guo, C., & Tibon, R. (2019). Unitization modulates recognition of within-domain and cross-domain associations: Evidence from event-related potentials. Psychophysiology, 56(11), e13446. 10.1111/psyp.13446 31369155 PMC6852485

[39] Liu, Z., Wang, Y., Zhu, Y., Yuan, J., & Liu, W. (2024). Improving associative memory in younger and older adults with unitization: Evidence from meta-analysis and behavioral studies. Frontiers in Aging Neuroscience, 16. 10.3389/fnagi.2024.1389957 PMC11153858 38846743

[40] Marsh, E. J. (2006). When does generation enhance memory for location? Journal of Experimental Psychology: Learning, Memory, and Cognition, 32, 1216–1220. 10.1037/0278-7393.32.5.1216 16938059

[41] Marsh, E. J., Edelman, G., & Bower, G. H. (2001). Demonstrations of a generation effect in context memory. Memory & Cognition, 29(6), 798–805. 10.3758/BF03196409 11716053

[42] McCurdy, M. P., & Leshikar, E. D. (2022). Contextual Framework of the Generation Effect. The American Journal of Psychology, 135(3), 251–270. 10.5406/19398298.135.3.01

[43] McCurdy, M. P., Viechtbauer, W., Sklenar, A. M., Frankenstein, A. N., & Leshikar, E. D. (2020). Theories of the generation effect and the impact of generation constraint: A meta-analytic review. Psychonomic Bulletin & Review, 27(6), 1139–1165. 10.3758/s13423-020-01762-3 32671573

[44] McDaniel, M. A., Waddill, P. J., & Einstein, G. O. (1988). A contextual account of the generation effect: A three-factor theory. Journal of Memory and Language, 27(5), 521–536. 10.1016/0749-596X(88)90023-X

[45] McFarland, C. E. Jr., Warren, L. R., & Crockard, J. (1985). Memory for Self-Generated Stimuli in Young and Old Adults1. Journal of Gerontology, 40(2), 205–207. 10.1093/geronj/40.2.205 3973362

[46] McGillivray, S., & Castel, A. D. (2010). Memory for age–face associations in younger and older adults: The role of generation and schematic support. Psychology and Aging, 25, 822–832. 10.1037/a0021044 21058867

[47] Memel, M., & Ryan, L. (2017). Visual integration enhances associative memory equally for young and older adults without reducing hippocampal encoding activation. Neuropsychologia, 100, 195–206. 10.1016/j.neuropsychologia.2017.04.031 28456521

[48] Morey, R. D., & Rouder, J. N. (2024). BayesFactor: Computation of Bayes Factors for Common Designs [Computer software]. R package version 0.9.12-4.7. Retrieved from https://CRAN.R-project.org/package=BayesFactor

[49] Mulligan, N. W., Lozito, J. P., & Rosner, Z. A. (2006). Generation and context memory. Journal of Experimental Psychology: Learning, Memory, and Cognition, 32, 836–846. 10.1037/0278-7393.32.4.836 16822151

[50] Naveh-Benjamin, M. (2000). Adult age differences in memory performance: Tests of an associative deficit hypothesis. Journal of Experimental Psychology: Learning, Memory, and Cognition, 26, 1170–1187. 10.1037/0278-7393.26.5.1170 11009251

[51] Naveh-Benjamin, M., Brav, T. K., & Levy, O. (2007). The associative memory deficit of older adults: The role of strategy utilization. Psychology and Aging, 22(1), Article 1. 10.1037/0882-7974.22.1.202 17385995

[52] Naveh-Benjamin, M., Hussain, Z., Guez, J., & Bar-On, M. (2003). Adult age differences in episodic memory: Further support for an associative-deficit hypothesis. Journal of Experimental Psychology: Learning, Memory, and Cognition, 29, 826–837. 10.1037/0278-7393.29.5.826 14516216

[53] Old, S. R., & Naveh-Benjamin, M. (2008). Differential effects of age on item and associative measures of memory: A meta-analysis. Psychology and Aging, 23, 104–118. 10.1037/0882-7974.23.1.104 18361660

[54] Parks, C. M., & Yonelinas, A. P. (2015). The importance of unitization for familiarity-based learning. Journal of Experimental Psychology: Learning, Memory, and Cognition, 41, 881–903. 10.1037/xlm0000068 25329077 PMC4404176

[55] Pesta, B. J., Sanders, R. E., & Nemec, R. J. (1996). Older Adults’ Strategic Superiority with Mental Multiplication: A Generation Effect Assessment. Experimental Aging Research, 22(2), 155–169. 10.1080/03610739608254004 8735150

[56] Pfister, R. (2021). Variability of Bayes Factor estimates in Bayesian Analysis of Variance. The Quantitative Methods for Psychology, 17(1), 40–45. 10.20982/tqmp.17.1.p040

[57] Quamme, J. R., Yonelinas, A. P., & Norman, K. A. (2007). Effect of unitization on associative recognition in amnesia. Hippocampus, 17(3), Article 3. 10.1002/hipo.20257 17203466

[58] Rhodes, S. M., & Donaldson, D. I. (2007). Electrophysiological evidence for the influence of unitization on the processes engaged during episodic retrieval: Enhancing familiarity based remembering. Neuropsychologia, 45(2), Article 2. 10.1016/j.neuropsychologia.2006.06.022 16930640

[59] Rhodes, S. M., & Donaldson, D. I. (2008). Electrophysiological evidence for the effect of interactive imagery on episodic memory: Encouraging familiarity for non-unitized stimuli during associative recognition. NeuroImage, 39(2), Article 2. 10.1016/j.neuroimage.2007.08.041 17950624

[60] Rue, H., Martino, S., & Chopin, N. (2009). Approximate Bayesian Inference for Latent Gaussian models by using Integrated Nested Laplace Approximations. Journal of the Royal Statistical Society Series B: Statistical Methodology, 71(2), 319–392. 10.1111/j.1467-9868.2008.00700.x

[61] Schönbrodt, F. D., & Wagenmakers, E.-J. (2018). Bayes factor design analysis: Planning for compelling evidence. Psychonomic Bulletin & Review, 25(1), Article 1. 10.3758/s13423-017-1230-y 28251595

[62] Shao, H., Opitz, B., Yang, J., & Weng, X. (2016). Recollection reduces unitised familiarity effect. Memory, 24(4), 535–547. 10.1080/09658211.2015.1021258 25793354

[63] Slamecka, N. J., & Graf, P. (1978). The generation effect: Delineation of a phenomenon. Journal of Experimental Psychology: Human Learning and Memory, 4, 592–604. 10.1037/0278-7393.4.6.592

[64] Squire, L. R., Knowlton, B., & Musen, G. (1993). The structure and organization of memory. Annual Review of Psychology, 44, 453–495. 10.1146/annurev.ps.44.020193.002321 8434894

[65] Taconnat, L., & Isingrini, M. (2004). Cognitive Operations in the Generation Effect on a Recall Test: Role of Aging and Divided Attention. Journal of Experimental Psychology: Learning, Memory, and Cognition, 30(4), 827–837. 10.1037/0278-7393.30.4.827 15238027

[66] Tibon, R., Ben-Zvi, S., & Levy, D. A. (2014). Associative Recognition Processes Are Modulated by Modality Relations. Journal of Cognitive Neuroscience. 10.1162/jocn_a_00586 24564465

[67] Tibon, R., Greve, A., & Henson, R. (2018). The missing link? Testing a schema account of unitization. Memory & Cognition, 46(7), 1023–1040. 10.3758/s13421-018-0819-3 29744769 PMC6711764

[68] Tibon, R., Gronau, N., Scheuplein, A.-L., Mecklinger, A., & Levy, D. A. (2014). Associative recognition processes are modulated by the semantic unitizability of memoranda. Brain and Cognition, 92, 19–31. 10.1016/j.bandc.2014.09.009 25463136

[69] Tibon, R., Vakil, E., Goldstein, A., & Levy, D. A. (2012). Unitization and temporality in associative memory: Evidence from modulation of context effects. Journal of Memory and Language, 67(1), 93–105. 10.1016/j.jml.2012.02.003

[70] Troyer, A. K., D’Souza, N. A., Vandermorris, S., & Murphy, K. J. (2011). Age-related differences in associative memory depend on the types of associations that are formed. Aging, Neuropsychology, and Cognition, 99999(1), Article 1. 10.1080/13825585.2011.553273 21390876

[71] Tu, H.-W., & Diana, R. A. (2021). The interaction of relational encoding and unitization: Effects on medial temporal lobe processing during retrieval. Behavioural Brain Research, 396, 112878. 10.1016/j.bbr.2020.112878 32890598 PMC7572763

[72] Verhaeghen, P., Geraerts, N., & Marcoen, A. (2000). Memory Complaints, Coping, and Well-Being in Old Age: A Systemic Approach. The Gerontologist, 40(5), 540–548. 10.1093/geront/40.5.540 11037932

[73] Yonelinas, A. P. (1997). Recognition memory ROCs for item and associative information: The contribution of recollection and familiarity. Memory & Cognition, 25(6), Article 6. 10.3758/BF03211318 9421560

[74] Yonelinas, A. P. (2002). The nature of recollection and familiarity: A review of 30 years of research. Journal of Memory and Language, 46(3), Article 3. 10.1006/jmla.2002.2864

[75] Yonelinas, A. P., Aly, M., Wang, W.-C., & Koen, J. D. (2010). Recollection and familiarity: Examining controversial assumptions and new directions. Hippocampus, 20(11), 1178–1194. 10.1002/hipo.20864 20848606 PMC4251874

[76] Yonelinas, A. P., Widaman, K., Mungas, D., Reed, B., Weiner, M. W., & Chui, H. C. (2007). Memory in the aging brain: Doubly dissociating the contribution of the hippocampus and entorhinal cortex. Hippocampus, 17(11), 1134–1140. 10.1002/hipo.20341 17636547 PMC2194291

[77] Zheng, Z., Li, J., Xiao, F., Broster, L. S., & Jiang, Y. (2015). Electrophysiological evidence for the effects of unitization on associative recognition memory in older adults. Neurobiology of Learning and Memory, 121, 59–71. 10.1016/j.nlm.2015.03.006 25858698

